# Interaction of V-type ATPase inhibitors and extracellular NAADP-triggered calcium release in skeletal muscle cells

**DOI:** 10.1186/1471-2210-11-S2-A24

**Published:** 2011-09-05

**Authors:** Florian Hiess, Martin Hohenegger

**Affiliations:** 1 Institute of Pharmacology, Center of Physiology and Pharmacology, Medical University of Vienna, 1090 Vienna, Austria

## Background

Nicotinic acid adenine dinucleotide phosphate (NAADP) has been identified as a calcium-mobilizing second messenger. NAADP is regularly enzymatically synthesized by ADP-ribosyl cyclases, in particular under acidic conditions. In nanomolar concentrations NAADP targets selectively the ryanodine receptor type 1 on the sarcoplasmic reticulum, and the two pore channels localized in dense-core secretory vesicles and lysosomes.

## Methods

Confocal microscopy was used to visualize calcium signalling and lysosomal movements within undifferentiated primary human skeletal muscle cells and C2C12 cells. Apoptosis and autophagy were analyzed by FACS, caspase 3 activity and Western blot analysis.

## Results

The application of extracellular NAADP to skeletal muscle cells resulted in a dose-dependent increase in cytosolic calcium transients. Within 180 seconds approximately 30% of the cells responded. The V-type ATPase inhibitors, bafilomycin A1 (Baf) and concanamycin A1 (Con), are widely used to inhibit NAADP-triggered calcium signals. However, by preventing lysosomal acidification calcium loading of these organelles is also inhibited. Accordingly, we determined calcium transients triggered by 100 nM Baf or Con. Interestingly, by the co-administration of extracellular NAADP with Baf or Con calcium transients were suppressed to basal level. The kinetics of lysosome destruction by Baf or Con were paralleled by “cell shrinking” and acidification. Beside these short-term effects, after 24 hours exposure caspase 3 activity and pre-G1 DNA fragmentation was already observed with 50 nM Baf. Conversely, autophagy was not induced.

## Conclusions

Hence, extracellular NAADP triggers calcium transients which were sensitive to Baf and Con. However, local changes in cytosolic pH and calcium concentrations may also result from lysosomal destruction induced by Baf or Con. Interestingly, longer incubation of skeletal muscle cells with Baf induced apoptosis with high potency. Thus, the application of V-type ATPase inhibitors in biological assays has to be carefully evaluated.

